# Metabolic engineering of the moss *Physcomitrella patens* to produce the sesquiterpenoids patchoulol and α/β-santalene

**DOI:** 10.3389/fpls.2014.00636

**Published:** 2014-11-18

**Authors:** Xin Zhan, Yu-Hua Zhang, Dong-Fang Chen, Henrik Toft Simonsen

**Affiliations:** ^1^Plant Biochemistry Laboratory, Department of Plant and Environmental Sciences, Copenhagen Plant Science Centre, University of CopenhagenCopenhagen, Denmark; ^2^Corporate R&D Division, Firmenich Aromatics (China) Co. Ltd.Shanghai, China

**Keywords:** *Physcomitrella*, sesquiterpenoids, fragrance, patchoulol, santalene

## Abstract

The moss *Physcomitrella patens*, has been genetically engineered to produce patchoulol and β-santalene, two valuable sesquiterpenoid ingredients in the fragrance industry. The highest yield of patchoulol achieved was 1.34 mg/g dry weight. This was achieved by non-targeted transformation of the patchoulol synthase and either a yeast or *P. patens HMGR* gene under the control of a 35S promoter. Santalene synthase targeted to the plastids yielded 0.039 mg/g dry weight of α/β santalene; cytosolic santalene synthase and 35S controlled *HMGR* afforded 0.022 mg/g dry weight. It has been observed that the final yield of the fragrance molecules is dependent on the expression of the synthase. This is the first report of heterologous production of sesquiterpenes in moss and it opens up a promising source for light-driven production of valuable fragrance ingredients.

## Introduction

Plant cells synthesize a diverse variety of specialized metabolites. These small organic molecules allow the plants to cope with various types of stress. These metabolites often have biological activities that are beneficial to humans, which make them of high commercial interest to biotechnological industry (Cragg et al., 2011). Many of these biologically active compounds are terpenoids, among theses sesquiterpenoids (C15 molecules) have benefited mankind as flavors, fragrances, pharmaceuticals, nutraceuticals, and industrial chemicals (Berger, 2007; Zwenger and Basu, 2008; Simonsen et al., 2013).

Biotechnological production is of special interest for sesquiterpenoids with known pharmacological properties or with application in foods and fragrances, since their limited availability in nature and the structural complexity of the molecules can make biotechnology the only commercial sustainable method of production (Daviet and Schalk, 2010; Simonsen et al., 2013). This however requires profound knowledge on the presence and biosynthesis of terpenoids in the original plant (Simonsen et al., 2009a; Weitzel and Simonsen, 2013). The antimalarial drug artemisinin is properly the sesquiterpenoid with most publications and work on biotechnological production. Recently the production have reached 25g/L of artemisinic acid in yeast and setting the goal for all other biotechnological production of sesquiterpenoids (Paddon et al., 2013).

Patchoulol and α/β-santalol are two sesquiterpenoids with profound woody notes that are used as fragrance ingredients in many perfumes. They are present in the essential oils of patchouli (*Pogostemon cablin*) and sandalwood (*Santalum album*). Due to the limited natural resources of the plants, the annual production of the essential oils can be very low and unstable leading to fluctuation in the price between 30–200 US dollar/kg for patchoulol and 1000–2000 US dollar/kg for santalol. This price fluctuation has afforded research into the biosynthesis of these valuable sesquiterpenoids and patchoulol synthase (PTS), α/β-santalene synthase (STS) and α/β-santalene monooxygenase (CYP76F39) were recently characterized and with this knowledge biotechnological production was initiated (Deguerry et al., 2006; Jones et al., 2011; Diaz-Chavez et al., 2013). For patchoulol the tobacco plant *Nicotiana tabacum* has been used for heterologously production and the final yield was 0.030 mg/g fresh weight (FW) (approximately 0.300 mg/g dry weight) in the leaves, along with a volatile emission of 50–100 ng/h/FW. One of the major improvements described in tobacco was the targeting of the sesquiterpenoid synthase to the plastids of tobacco. This afforded a 40,000 fold increase in yields (Wu et al., 2006), thus was also tested here. Biotechnological production of santalene has been performed in yeast, and afforded 0.086 mg/g santalene per biomass (Scalcinati et al., 2012).

As with other attempts of biotechnological production of sesquiterpenoids, e.g., in yeast (Ro et al., 2006) up-regulation of the key step in cytosolic terpenoid pathway, the HMGR, was attempted. It has been demonstrated that by using a version where the regulatory parts of the gene is removed through truncation, up to 75% increase in production can be achieved (Ro et al., 2008). The use of truncated HMGR was also performed here. HMGR is the key regulatory enzyme in the MVA pathway in both plants and yeast, and expression studies in plants have shown that regulation is on both the transcription and the posttranscriptional level, but the genes encoding all enzymes in the pathways MVA and MEP are not tightly co-regulated. This suggest that up regulation should be done on all the individual enzyme in order to obtained the highest flux of carbon through to the precursors of the desired terpenoid, here farnesyl diphosphate (FPP) (Vranova et al., 2013).

The moss *Physcomitrella patens*, a non-vascular plant, has been proposed as a novel production host to produce sesquiterpenoids and diterpenoids in recent years (Anterola et al., 2009; Simonsen et al., 2009a; Bach et al., 2014). *P. patens* itself produces large amounts (approximately 3 mg/g d.w.) of the tetracyclic diterpene *ent*-16-α-hydroxy-kaurene, which reaches concentrations about one-third that of chlorophyll a and b (Von Schwartzenberg et al., 2004). In 2006, the bi-functional diterpene synthase gene named copalyl pyrophosphate/kaurene synthase (*CPS/KS*) gene was cloned from *P. patens*. The characterization showed that enzyme afford two diterpenoid products *ent*-kaur-16-ene and *ent*-16-α-hydroxy-kaurene (Hayashi et al., 2006). *CPS/KS* is the only active terpene synthase found in the genome of *P. patens*. Thus, establishing a knock out of this gene might afford higher yields of other terpenoids if this would not show any severe reduction of the growth of the protonemal tissue or other effects with a negative influence on the final production of sesquiterpenoids.

Transformation of *P. patens* unlike other plants can utilize both homologous recombination and non-targeted transformation (Bach et al., 2014). In this study, non-targeted transformation has been used utilizing already established in-house vectors.

Here we report the production of two valuable sesquiterpenoids for the fragrance industry patchoulol and β-santalene in the moss *P. patens*. This is the first report on heterologous production of sesquiterpenoids in *P. patens* along with some attempts to increase the production.

## Materials and methods

### The moss cultivation and maintenance

Wild type *P. patens* (Gransden ecotype) was obtained from the International Moss Stock Center at the University of Freiburg (http://www.moss-stock-center.org/). This was propagated on PhyB agar plates or in PhyB liquid media under standard condition for *P. patens* cultivation (Cove et al., 2009). For sub-culturing and maintenance of wild type and transformed lines *P. patens* tissue was blended using a Polytron tissue homogenizer (PT 1200E, Kinematic AG) and distributed evenly onto new PhyB agar plates (Cove et al., 2009).

### Vectors construction

The cloning vector pJET1.2 (Thermo Scientific) was used as the backbone for construction of expression vectors for moss transformation. Two USER (Uracil-Specific Excision Reagent) compatible binary vectors pCAMBIA1300Su and 2300Su were both double-digested by PsiI and PmeI (New England Biolabs) (Nour-Eldin et al., 2006). The digested blunt-end DNA fragments containing the antibiotic resistant cassettes and the USER cloning sites were ligated into the pJET1.2 backbone and the constructed vectors were named pUNI33 and pUNI6, respectively.

The two genes patchoulol synthase gene (*PTS*) from *P. cablin* and α/β-santalene synthase gene (*STS*) from *S. album* were provided by Firmenich and amplified with USER-compatible overhangs by the primer pairs PTS-F&R and STS-F&R. They were subsequently integrated into pUNI33 using USER cloning techniques and named pUNI33 PTS and pUNI33 STS (Nour-Eldin et al., 2006).

For overexpression of truncated *HMGR* in *P. patens* the *PpHMGR* was obtained from the cosmoss database (www.cosmoss.org). The catalytic domains were predicted by THHMM Server v. 2.0 (http://www.cbs.dtu.dk/services/TMHMM/) and compared with the truncated HMGR version from yeast. This yielded a truncated version identified as *PptHMGR*. This and truncated *S. cerevisiae HMGR* gene (Ro et al., 2006) were amplified using the primer pairs PptHMGR-F&R and SctHMGR-F&R and integrated into pUNI6 and named pUNI6 PptHMGR and pUNI6 SctHMGR.

A *P. patens* lines knocked out in the *CPS/KS* gene was obtained using a previously published plasmid pCL755 that was linearized and through homologous recombination lead to a knock out of the *CPS/KS* gene (Zhan et al., in press).

Plastidic targeting was obtained through adding the *Arabidopsis* RuBisCO small subunit transit peptide to the genes of interest. For plastidic targeting of the *PTS* gene, the tp*PTS* fragment was amplified using the primer pair tp-F & PTS-R from pBDON tpPTS-tpFPS (provided by Firmenich) with overhangs for USER cloning and inserted into pUNI33 (named pUNI33 tpPTS afterwards) (Wu et al., 2006). To construct the tp*STS* fragment, the transit peptide sequence from the pBDON tpPTS-tpFPS construct and the *STS* gene was amplified individually by primer pair's tp-F & tp-R and tpSTS-F & STS-R with USER overhangs. They were subsequently fused together and cloned into pUNI33 by USER fusion (named pUNI33 tpSTS) (Nour-Eldin et al., 2010). All the information about the primers and vectors used are listed in Table S1 and S2.

The pfuX7 DNA polymerase was used in all the PCR reactions and the classical 3-step PCR program was used to amplify the DNA fragments used for subsequent USER cloning techniques as previously described (Nørholm, 2010).

### PEG-mediated moss transformation and selection

Before PEG-mediated transformation, all vectors were linearized using NotI-HF (New England Biolabs), concentrated by isopropanol and approximately 20 μg of DNA was prepared, except pCL755, which was double digested by EcoRI and NdeI. Linearized DNA was then mixed with moss protoplasts using the PEG-mediated transformation method previously described (Cove et al., 2009). After 5–7 days of regeneration, the moss tissue was moved to antibiotic selection to recover stable transgenic lines using the appropriate antibiotics (Bach et al., 2014). Stabled transgenic lines were recovered after two rounds of antibiotic selection, and are kept as protonemal tissue through sub culturing of the stable lines.

### Volatile metabolite profiling

The volatile metabolite profile of all the transgenic lines was determined by HS-SPME (Headspace-Solid Phase Micro-Extraction) and GC-MS (Gas Chromatography-Mass Spectrum) analysis that has been shown to be very useful technique for volatile plant metabolites (Drew et al., 2012).

For fast and qualitative measurements of the newly transformed lines, these where grown in 20 ml GC vials on solid PhyB media. This allow for the use of autosampler for handling of a large number of lines using HS-SPME GC-MS for analysis of the volatiles. The HS-SPME GC-MS was performed as previously described (Simonsen et al., 2009b; Bach et al., 2014), shortly the fiber used for HS-SPME was Supelco 27298-U, 50/30 μm divi-nylbenzene-carboxen-polydimethylsiloxane-stableFlex/SS (1cm). The fiber was injected 5 cm in to the vial to collect the HS with 1 cm of the fiber exposed. The extraction time was 60 min without agitation. The fiber was directly transferred to the injection port after sampling with desorption time of 2 min. Fibers were reconditioned for 10 min in a needle-oven at 270°C. The GC-MS analysis was performed on a Shimadzu—-GCMS-QP2010 plus (GC-2010) with a CTC autosampler AOC-5000, equipped with non-cooled tray for 20 ml vials. The injection temperature was set for 250°C. The column used was a Supelco 28466-U, SLB-TM 5ms, fused Silica capillary column 15 m, 0.1 mm diameter × 0.1 μm film thickness inserted directly into the ion source of the MS. Driving gas was He (99.9999%). Using direct injection and pressure control mode the pressure was kept at 162.1 kPa giving a column flow of 0.15 mL min^−1^. The oven temperature was set at 45°C for 3 min, then increased to 270°C with a rate of 10° min^−1^ and maintained for 8 min. The ion source temperature was 260°C with an interface temperature 250°C. The total run time was 34 min. The ionization electron energy was 70 eV and the mass range scanned was 40–600 Da in full scan acquisition mode. For analysis of essential oils, a solvent cut was set at 8 min. All data were analyzed using the Shimadzu software Lab Solutions, GCMS Solutions Ver 2.50 SU3, using the latest libraries provided by NIST05 and Wiley (Wiley 8.0) including our own data references. Obtained spectra were compared with the spectra in the mass spectral libraries. Compounds were identified comparing the data with library information of MS and retention indices (*I*). All reference *I*s were drawn from www.pherobase.com in April 2014, and based on reference within, references were checked for all compounds. Patchoulol and α-santalene standards was obtained from Firmenich.

### Volatile metabolite quantification

Quantification of volatile metabolites was performed as described previously (Bach et al., 2014). Fresh moss tissues were blended and inoculated (each inoculum was approximately 10 mg d.w.) into 50 ml PhyB medium in 250 ml shake flasks and cultivated on a rotary shaker in the standard conditions. After 2 weeks, the media and moss tissues were extracted using 50 (for the PTS lines) or 25 ml heptane (for the STS lines) by hand shaking for 1 min. After phase separation, 1 ml of the organic phase was taken into a GC vial and 1 μl was injected into the GC-MS. Quantification was achieved based on a standard curve from authentic standards (patchoulol and α-santalene). Meanwhile, the moss tissue was filtered using a vacuum pump and dried at 60°C overnight and the dry weights were measured the next day. GC-MS analysis was performed on a GCMS 7890/5975C (Agilent) equipped with a LTM column module (DB-1MS) (10 m × 0.18 mm i.d. × 0.18 μm). The samples (1 μl) were injected with a split ratio of 25:1 into LTM (DB-1MS) column using the following temperature program: 50°C (held for 1 min), 50–320°C (30°C /min, held for 1 min), and the total time is 11 min (2 min solvent delay). The oven temperature was 200°C (held for 11 min). The injector temperature of GC was 250°C. The ion source temperature of the mass spectrometer was 230°C and the transfer line temperature was set at 250°C. Helium was used as carrier gas at a constant flow rate of 0.7 ml/min. Data were acquired by EI+ with SIM (Selected Ion Monitor) mode. The detail of the SIM method to quantify patchoulol and α-santalene was as follows: RT (retention time) 2.00–6.00 min diagnostic ions 98, 138, 161, 222 were selected to monitor patchoulol and 93, 94, 107, 121, 122, 204 for α/β-santalene. For *ent*-16-α-hydroxy-kaurene [(4aR,6aS,8R,9R,11aR,11bR)-4,4,8,11b-tetramethyltetradecahydro-6a,9-methanocyclohepta-α-naphthalen-8-ol], RT 6.00–11.00 min 232, 257, 272, 290 were selected as diagnostic ions. *Ent*-16-α-hydroxy-kaurene was quantified in approximate level according to the external standard (patchoulol or α-santalene) curves. The statistical significance was calculated using Student's *t*-test defining the significant level as *P* value < 0.05. Data are given in Table 1.

**Table 1:** **Production levels of patchoulol and α/β-santalene in the different moss lines, along with *ent*-16- α-hydroxy-kaurene, stigmasterol, chlorophyll, lutein and carotene**.

	**Patchoulol**	**α-santalene**	**β-santalene**	***ent*-16-α-OH-kaurene**	**Stigmasterol**	**Chlorophyll a**	**Chlorophyll b**	**Lutein**	**β-carotene**
WT				2.42 ± 0.10	1.83 ± 0.09	27.2 ± 5.63	5.57 ± 0.99	2.39 ± 0.34	2.65 ± 0.43
KO				0	1.54 ± 0.03	33.3 ± 3.86	7.73 ± 0.89	2.97 ± 0.35	3.36 ± 0.41
WT-PTS10	0.29 ± 0.04			3.23 ± 0.07	1.70 ± 0.30				
WT-PTS14	0.20 ± 0.10			0.16 ± 0.11	1.70 ± 0.20				
WT-PTS19	0.83 ± 0.08			0.61 ± 0.14	1.61 ± 0.08				
WT-PTS27	0.32 ± 0.04			0.39 ± 0.13	1.02 ± 0.06				
WT-PTS35	0.60 ± 0.04			0.22 ± 0.03	1.38 ± 0.26	24.1 ± 1.43	5.98 ± 0.32	2.09 ± 0.13	2.45 ± 0.26
PTS35-S16	1.33 ± 0.36			0.57 ± 0.17	2.13 ± 0.07				
PTS35-S36	1.00 ± 0.24			0.66 ± 0.13	1.87 ± 0.17				
PTS35-S39	1.34 ± 0.04			0.43 ± 0.09	1.79 ± 0.15				
PTS35-KO	0.38 ± 0.03			0	2.04 ± 0.07	31.5 ± 1.83	8.03 ± 0.32	2.81 ± 0.09	2.76 ± 0.09
WT-tpPTS2	0.02 ± 0.01			1.54 ± 0.14	2.00 ± 0.22	29.2 ± 5.86	6.57 ± 1.22	2.58 ± 0.56	3.05 ± 0.56
WT-STS3		ND	ND	3.21 ± 0.55	1.52 ± 0.07				
WT-STS6		ND	ND	1.69 ± 0.37	1.23 ± 0.13				
WT-STS11		ND	ND	0.04 ± 0.02	1.55 ± 0.21				
WT-STS13		ND	ND	1.51 ± 0.50	1.21 ± 0.11				
STS6-P7		0.022 ± 0.011	0.020 ± 0.010	0.850 ± 0.094	1.43 ± 0.38				
STS6-P12		0.005 ± 0.000	0.005 ± 0.000	0.075 ± 0.000	1.90 ± 0.34				
STS6-P14		0.010 ± 0.002	0.009 ± 0.002	0.409 ± 0.039	1.35 ± 0.06				
STS6-P19		0.003 ± 0.001	0.003 ± 0.001	0.132 ± 0.010	1.30 ± 0.16				
STS6-P20		0.005 ± 0.000	0.004 ± 0.000	0.520 ± 0.006	1.39 ± 0.10				
STS6-S17		0.005 ± 0.001	0.005 ± 0.002	0.478 ± 0.044	2.13 ± 0.17				
STS6-S18		0.006 ± 0.001	0.005 ± 0.001	0.442 ± 0.115	2.09 ± 0.48				
STS6-S19		0.006 ± 0.001	0.005 ± 0.001	0.344 ± 0.014	2.32 ± 0.28				
STS6-S29		0.005 ± 0.001	0.004 ± 0.001	0.129 ± 0.010	1.61 ± 0.24				
WT-tpSTS1		0.039 ± 0.008	0.035 ± 0.005	16.557 ± 5.034	1.17 ± 0.05	39.1 ± 0.71	7.84 ± 0.29	3.37 ± 0.25	3.88 ± 0.09

*WT, the wild type line; KO, PpCPS/KS knockout line; WT-PTS/STS, cytosolic patchoulol synthase (PTS)/santalene synthase (STS) lines; PST35/STS6-Sx, SctHMGR-overexpressing PTS/STS lines; STS6-Px, PptHMGR-overexpressing STS lines; PTS35-KO, PpCPS/KS knock out in WT-PST35; WT-tpPTS/tpSTS, plastid targeted PTS/STS lines in WT background; ND, Not Detectable. Unit, mg/g d.w*.

### Non-volatile metabolites quantification

Five non-volatile metabolites in the terpenoid biosynthetic pathway that all are part of the general plant metabolites, stigmasterol, chlorophyll a+b, β-carotene and lutein were quantified using external authentic standards. The statistical significance was calculated using Student's *t*-test defining the significant level as *P* value < 0.05.

For stigmasterol measurement, 2-week old moss tissue of each line was freeze-dried overnight and dissolved in 2 ml dichloromethane:methanol (1:1) and vortexed for 2 h. The solution were transferred into a 2 ml centrifuge tube and centrifuged at 4400 rpm for 5 min. The organic phase were subsequently filtered and 1 μl of the extract was injected into a GC-FID for analysis. The quantification was performed by Agilent GC-FID (6890) with a LTM column module. GC method: column DB-1MS 10 m, 0.18 mm ID, 0.18 μm film, 0.7 ml/min He constant flow, injector 250°C, oven: 150°C, 1 min; 150–340°C, 10°C/min, 340°C 2 min.

For the measurement of pigments, 2-week old moss tissue was freeze dried overnight and soaked in 30 ml acetone/H_2_O/25% NH_4_OH (80/19/1) and blended using the tissue homogenizer to disrupt the dry biomass. The samples were then vortexed for 1 h and an aliquot of the solution was transferred into 1.5 ml centrifuge tube and centrifuged at 4400 rpm for 5 min. The organic phases were subsequently transferred into HPLC vials and 15 μl was injected into an Agilent High-Performance Liquid Chromatography 1200 series with a photodiode array detector. The column was a Waters Atiantis® T3 (2.1 × 150 mm, 3 μm) set at 35°C. The solvent system consisted of two solvents: A: acetonitrile/methanol/water (84:9:7), and B: methanol/ethyl acetate (68:32). Both solvents contained 0.1% triethylamin. Samples was eluted with a flow rate of 0.8 ml/min with a linear gradient from 100% A to 100% B over 20 min. This was followed by an isocratic elution with 100% B for 5 min, and a linear gradient of 100% B to 100% A in 1 min, then the column was equilibrated with 100% A for 9 min set to 1 ml/min.

### Transcript analysis of selected terpenoid genes

The terpene synthase genes PTS and STS, along with the regulatory gene HMGR in the cytosolic part of the terpenoid biosynthesis, were selected for transcript analysis. Only the isogenes with the most abundant EST evidences were selected for transcripts level study according to the cosmoss database (www.cosmoss.org). The transcripts were quantified by RT-qPCR (Real Time-quantitative PCR). Three biological replicates were selected for each transgenic line 2 weeks following subculture and their total RNAs were extracted using Spectrum™ Plant Total RNA Kit (Sigma) and the concentrations and quality (A260/A280 > 1.8; A260/A230 > 2.0) were measured using Nanodrop 1000D. The first-strand cDNA was subsequently generated using iScript™ cDNA synthesis Kit (Bio-Rad). The cDNA was then used as templates in RT-qPCR using the dyNAmo SYBR Green qPCR kit (Thermo Scientific). *P. patens actin2* (NP_188508) was used as the reference gene here and the transcripts level was calculated using the 2^−ΔΔ*t*^ method (Livak and Schmittgen, 2001). The nucleotide sequences of the qPCR primers are listed in Table S3. The statistical significance was calculated using Student's *t*-test defining the significant level as *P* value < 0.05.

## Results

*P. patens* was engineered to produce patchoulol, α- and β-santalene by non-targeted transformation of *PTS* and *STS*. Additionally, the yields of patchoulol, α- and β-santalene were improved by *HMGR* overexpression under the control of the 35S promoter. The yield of α- and β-santalene was also improved in one transgenic line where STS was targeted to the chloroplasts. However, disruption of the only functional endogenous terpene synthase in *P. patens CPS/KS* gene did not improve, but rather reduced the production levels of patchoulol, α- and β-santalene.

### Volatile profiles of *PTS* expressing *P. patens* lines

**Figure 1:**
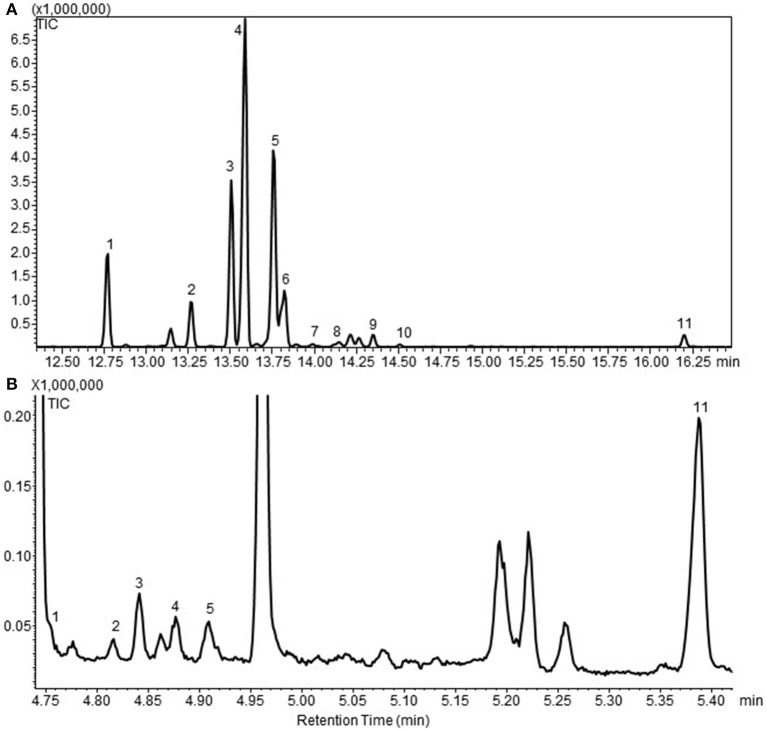
**(A)** A gas chromatogram of the sesquiterpenoid profile of cytosolic patchoulol lines detected in the headspace of the moss lines by GC-MS. **(B)** A representative gas chromatogram of the sesquiterpenoid profiles of patchoulol lines. This chromatogram is obtained from the quantitative analysis of patchoulol lines in liquid culture followed by liquid extraction. The GC-MS program and equipment is different from the one used in all the headspace analysis'. This chromatogram clearly show that patchoulol is the major product. The compounds was identified as 1. β-patchoulene; 2. β-caryophyllene; 3. α-guaiene; 4. seychellene; 5. α-patchoulene; 6. γ-patchoulene; 7. guai-4,11-diene; 8. α-selinene; 9. δ-guaiene; 10. α-panasinsen; 11. patchoulol. The identification was based on the mass spectra and retention index (RI) of the compounds, and the authentic standard patchoulol are shown in Figure S1.

To generate cytosolic PTS lines, wild type (WT) moss were transformed with the linearized vector pUNI33 PTS by non-targeted transformation. After two rounds of antibiotic selection, 44 independent stable lines named WT-PTS1 to WT-PTS44 were obtained. Of these, 29 emitted patchoulol and other sesquiterpene products was analyzed by HS-SPME-GCMS (Figure 1A). Eleven sesquiterpenoids were identified in the headspace in addition to the endogenous diterpenoids (Hayashi et al., 2006; Zhan et al., in press), being β-patchoulene (1), β-caryophyllene (2), α-guaiene (3), seychellene (4), α-patchoulene (5), γ-patchoulene (6), guai-4,11-diene (7), α-selinene (8), δ-guaiene (9), α-panasinsen (10), and patchoulol (11). In the headspace, it was observed that seychellene was the most abundant followed by α-patchoulene and α-guaiene. Using liquid extraction patchoulol was found to be the major sesquiterpenoid product (Figure 1B) along with other sesquiterpenoids, this is in line with previous findings (Deguerry et al., 2006).

**Figure 2:**
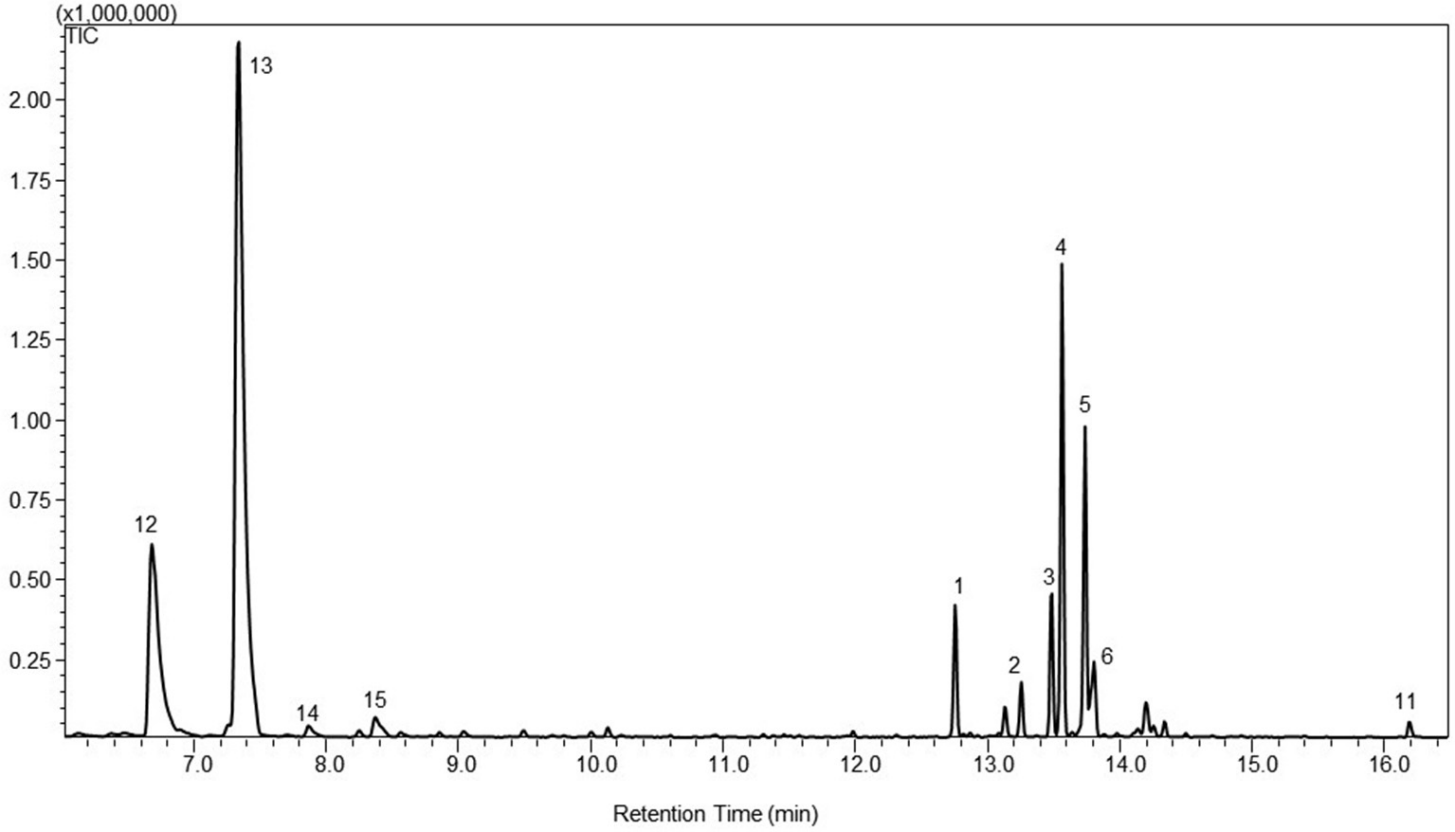
**A representative gas chromatogram of the mono- and sesquiterpenoid profile of patchoulol lines with patchoulol synthases targeted to the plastids, WT-tpPTS2**. The chromatogram show the detected compounds in the headspace of the moss lines using GC-MS. The compounds was identified as 12. β-myrcene; 13. limonene; 14. γ-terpinene; 15. α-terpinolene; 1. β-patchoulene; 2. β-caryophyllene; 3. α-guaiene; 4. seychellene; 5. α-patchoulene; 6. γ-patchoulene; 11. patchoulol. The identification was based on the mass spectra and retention index (RI) of the compounds, and the authentic standard patchoulol are shown in Figure S1.

The PTS enzyme was also targeted into the chloroplasts of *P. patens* by fusing the transit peptide of *Arabidopsis* RuBisCO small subunit to the N-terminus of the enzyme (Wu et al., 2006). Wild type *P. patens* and PpCPS/KS KO line were used as the background lines to test the effect of the plastidic targeting of the PTS enzyme on patchoulol production. Besides the native diterpenoids and the heterologous sesquiterpenoid products found in the cytosolic PTS lines, the headspace analysis of the plastidic PTS lines showed the emission of the monoterpene products β-myrcene (12), limonene (13), γ-terpinene (14), and α-terpinolene (15) (Figure 2).

### Quantification of patchoulol in transgenic *P. patens* lines

Five lines expressing *PTS*, WT-PTS10, 14, 19, 27 and 35, were selected according to their relatively larger peak area of seychellene in the headspace for metabolite quantification. Quantification of the sesquiterpenoids showed that the five WT-PTS lines produced 0.2–0.8 mg patchoulol/g dry weight (d.w.) (Table 1).

In order to increase the yield of patchoulol truncated versions of HMGR was introduces into a *PTS* expressing moss. One of the best producers, WT-PTS35 was selected as the background line for this experiment. In order to avoid the post-translational regulation (Chappell et al., 1995) the N-terminal truncated *HMGR* from *Saccharomyces cerevisiae* (*SctHMGR*) was used for overexpression. In the *SctHMGR* (truncated *S. cerevisiae HMGR*)-overexpressing PTS lines, three of the 39 obtained transgenic lines named PTS35-S16, PTS35-S36, and PTS35-S39 were selected according to the peak areas of seychellene observed in headspace for the subsequent metabolite quantification. The best line afforded a 2-fold increase of the patchoulol level compared to the background line. The overall yield was up to 1.34 mg/g d.w. of patchoulol in PST35-S39 after 2 weeks of cultivation. This is highest yield obtained of patchoulol, and these lines have a profound scent of patchoulol during cultivation.

*P. patens* have one functional endogenous terpene synthase gene *PpCPS*/*KS* (Hayashi et al., 2010; Zhan et al., in press). In order to increase the levels of patchoulol a knock-out of this synthase in the patchoulol producing line WT-PTS35 was established as previously described (Zhan et al., in press). This afforded a 40% lower yield than the background with a total of 0.38 mg/g d.w. The molecular explanation for this effect needs further studies, but results indicate that this strategy is challenging.

The patchoulol producing line, where PTS was targeted to the plastids only afforded a total yield of 0.02 mg/g after 14 days of cultivation, which is different from what was previously observed (Wu et al., 2006).

### Volatile profiles of *STS* expressing *P. patens* lines

**Figure 3:**
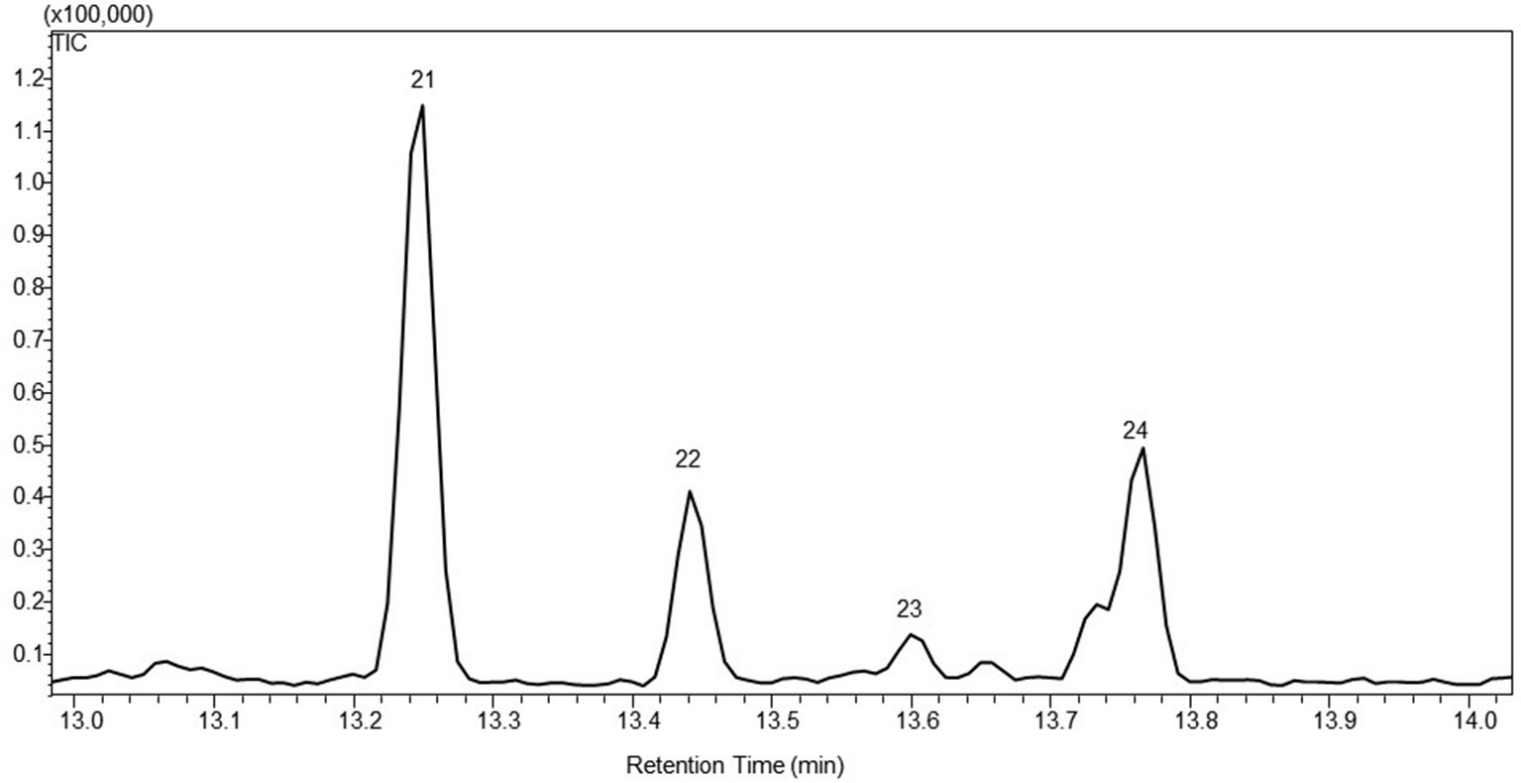
**A representative gas chromatogram of the sesquiterpenoid profile of santalene producing lines**. The chromatogram show the detected compounds in the headspace of the moss lines using GC-MS. The compounds was identified as 21. α-santalene; 22. α-bergamotene; 23. epi-β-santalene; 24. β-santalene. The identification was based on the mass spectra and retention index (RI) of the compounds, and the authentic standard patchoulol are shown in Figure S1.

Santalene synthase gene was introduced into *P. patens* using the same approach as with *PTS*. This afforded 15 independent transgenic lines after two rounds of antibiotic selection. Four, named WT-STS3, 6, 11 and 13, showed emission of α-santalene (21), α-bergamotene (22), epi-β-santalene (23) and β-santalene (24) into the headspace besides the endogenous diterpenoid metabolites (Figure 3). The co-expression of truncated *HMGR* genes did not change the volatile profile, but significantly enlarge the amount of observed sesquiterpenoids.

**Figure 4:**
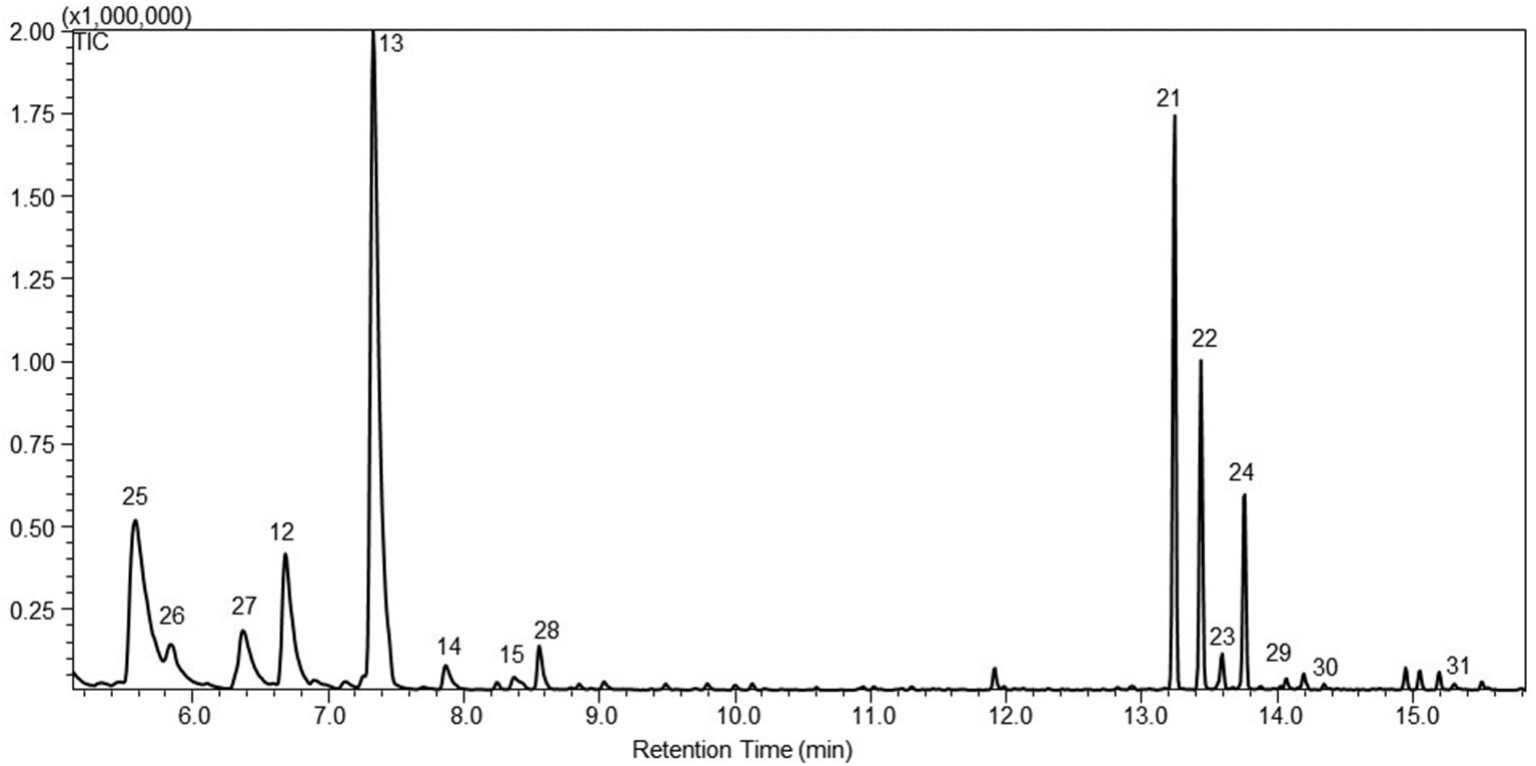
**A representative gas chromatogram of the mono- and sesquiterpenoid profile of santalene lines with santalene synthases targeted to the plastids, WT-tpSTS1**. The chromatogram show the detected compounds in the headspace of the moss lines using GC-MS. The compounds was identified as 25. α-pinene; 26. camphene; 27. β-pinene; 17. β-myrcene; 18. limonene; 19. γ-terpinene; 20. α-terpinolene; 28. linalool; 21. α-santalene; 22. α-bergamotene; 23. epi-β-santalene; 24. β-santalene; 29. (E)-β-farnesene; 30. β-bisabolene; 31. α-santalan-10-ol. The identification was based on the mass spectra and retention index (RI) of the compounds, and the authentic standard patchoulol are shown in Figure S1.

As with PTS, STS was targeted to the plastids of *P. patens* using the same approach. Four plastidic STS lines were obtained and headspace analysis showed that one of the four lines, named WT-tpSTS1, emitted both sesquiterpenoids and monoterpenoids (Figure 4). The monoterpenoids were identified as α-pinene (25), camphene (26), β-pinene (27), β-myrcene (17), limonene (18), γ-terpinene (19), α-terpinolene (20), and linalool (28). Three additional sesquiterpenoids, (E)-β-farnesene (29), β-bisabolene (30), and α-santalan-10-ol (31) were also detected in this plastidic line, which were not observed in the cytosolic lines (Figure 4).

### Quantification of α/β-santalene in transgenic *P. patens* lines

The yield of α- and β-santalene could not quantified in the four cytosolic WT-STS lines (Table 1), even though α- and β-santalene was identified in the headspace.

In order to increase the yields of α- and β-santalene the line WT-STS6 was transformed with N-terminal truncated versions of *HMGR* from either moss *P. patens* (*PptHMGR*) or yeast *S. cerevisiae* (*SctHMGR*). Ten transgenic STS lines with *PptHMGR* or *SctHMGR* overexpression were obtained and the yields of α- and β-santalene after 14 days of cultivation were found to be in the range of 0.003–0.022 mg/g d.w. (Table 1).

STS was also targeted into the chloroplasts. This afforded 0.039 and 0.035 mg/g d.w. of α- and β-santalene in WT-tpSTS, which is the highest α- and β-santalene yield achieved in *P. patens* (Table 1).

### General terpenoid metabolite levels in the transgenic lines

*Ent*-16-α-hydroxy-kaurene was decreased by introduction of the heterologous terpenoid synthases in the majority of the established lines. In WT-PTS19, 27, 35, and WT-STS11 the amount of *ent*-16-α-hydroxy-kaurene was significantly reduced compared to the WT moss (*P* < 0.05) (Table 1), only the level of *ent-16*-α-hydroxy-kaurene in WT-tpSTS1 was significantly increased compared to WT.

Overexpression of *SctHMGR* in WT-PTS35 did increase the amounts of *ent*-16-α-hydroxy-kaurene by 2–3 folds relative to the background, but this was still lower than in the WT. Overexpression of *PptHMGR* or *SctHMGR* in WT-STS6 further reduced the amounts of *ent*-16-α-hydroxy-kaurene (Table 1). The reduction of diterpene levels suggest that isopentenyl diphosphate levels in the plastids are affected by the levels and use of isopentenyl diphosphate in the cytosol.

Stigmasterol, the primary sterol constituent in *P. patens* (Morikawa et al., 2009), was quantified for all the transgenic lines. It was decreased in all transgenic lines expressing either *PTS* or *STS* in WT moss background, except the plastidic PTS line WT-tpPTS2 (Table 1). The strong reduction of stigmasterol in these lines might correlate with production of new sesquiterpenoids, since they used the same precursor FPP. For all the lines with overexpression of *PptHMGR* or *SctHMGR*, an increase in stigmasterol level was observed when compared to the background lines (Table 1) again suggesting that more FPP also affect the levels of sterols. When compared to WT, the amounts of stigmasterol were even higher in some lines, which indicate an overall increased metabolic flux through the MVA pathway.

The pigments chlorophyll a and b, lutein and β-carotene were also quantified in five of the PTS/STS lines (Table 1), but no significant change was observed for any of the transgenic lines.

### Analysis of transcripts related to terpenoid biosynthesis

In order to test the correlation between the enzyme expression levels and chemical profiles of the transgenic moss lines and improve the understanding of terpenoid metabolism in *P. patens*, the terpene synthases and HMGR's were analyzed for their transcripts level change by RT-qPCR (Vranova et al., 2013). The results are given in Table 2. The expression levels of the terpene synthases and the significant change of HMGR might explain change in the levels of the desired product in the individual lines, though the use of actin as reference gene increase the error margin of the analysis. Especially PTS35-S16, 36, and 37 have a combination of high level of PTS and HMGR overexpression and high level of production of patchoulol.

**Table 2:** **Expression levels of patchoulol synthase gene (*PTS*), santalene synthase gene (*STS*) and hydroxy-methylglutaryl reductase (*HMGR*) in the different mutant lines given in ΔCT-values**.

	**WT**	**KO**	**WT-PTS10**	**WT-PTS14**	**WT-PTS19**	**WT-PTS27**	**WT-PTS35**	**PTS35-S16**	**PTS35-S36**	**PTS35-S39**	**WT- tp PTS2**	**PTS35-KO**	**WT-STS6**	**WT-STS13**	**WT- tp STS1**	**KO-STS1**	**STS6-P7**	**STS6-P12**	**STS6-P14**	**STS6-S17**	**STS6-S18**	**STS6-S29**
*PTS*/*STS*			9.16	3.17	11.55	22.04	5.73	52.02	17.42	44.71	2.99	2.46	0.01	0.01	6.67	0.08	0.01	0.00	0.00	0.01	0.02	0.01
*SctHMGR*								1.87	0.63	0.50										0.00	0.00	0.05
Total *PpHMGR*																	5.71^*^	7.79^*^	5.40^*^			
Native *PpHMGR*	1.00	1.68	1.49	4.20^*^	2.74	3.34	3.01	5.97^*^	6.16^*^	6.16^*^	0.77	2.21	1.65	3.73	4.53^*^	2.07	2.51^*^	2.98^*^	3.21^*^	1.90	2.27	2.62

*Actin was used as reference gene. The primers used for the qPCR is found in Table S3*.

* *Indicates where the mutant is significantly different from the background line based on a student t-test*.

## Discussions

Efforts to develop microbial platforms for terpenoid production, especially the yeast *S. cerevisiae*, have been ongoing for more than 20 years (Asadollahi et al., 2009, 2010; Paddon et al., 2013). By combining the rational genetic engineering of yeast and optimization of the fermentation process, a commercially-relevant high titer of 25 g/L of artemisinic acid has been achieved, a sesquiterpene precursor to the antimalaria drug artemisinin (Paddon et al., 2013).

*P. patens* clearly shows a potential to become an efficient producer of terpenoids. With only a few modification and no culture optimization, we afforded a total yield of patchoulol of 1.34 mg/g d.w. in a 50 ml culture making it ca. 1.00 mg/L, even though large differences was observed in the various stable lines produced.

In all the STS lines, the yields of α- and β-santalene were far below the ones of patchoulol in the PTS lines, although the same expression vectors and overexpression strategies was used. The examination on the transcripts level of the STS lines revealed the extremely low expression levels of the STS gene, which could explain the low yields of α- and β-santalene. The observed expression levels are based on actin. Actin is not the best choice of reference gene, but it is estimated that the error introduced by the use of actin cannot explain the difference in expression between the two heterologous terpene synthase (Pan et al., in press). The molecular mechanism leading to the low expression of the STS gene is unknown, but positional effects introduced during transformation along with unknown transcriptional regulations could be the explanations. If the low expression level can be overcome, e.g., by using homologs recombination into neutral locus's, or by using versions of *STS* where the codon usages is optimized for moss expression this could lead to higher production levels of α- and β-santalene. The use of codon-optimized genes to increase translation have previously been shown to be very useful (Stenøien, 2004, 2007), whereas understanding of positional effects in the moss genome is much less understood.

HMGR catalyzes the key regulatory step in the MVA pathway (Vranova et al., 2013), thus is the first enzyme to be up regulated in terpenoid production systems (Asadollahi et al., 2010; Paddon et al., 2013). The yield of patchoulol was increased by 1.7 to 2.2-fold and stigmasterol by 1.3 to 1.5-fold upon the overexpression of a truncated yeast *HMGR* (*SctHMGR*) in WT-PTS35 (PTS-S16, 36 and 39, Table 1). The introduced *SctHMGR* only showed very low expression, but led to an up-regulation of the endogenous *HMGR* gene that also would be a benefit for downstream biosynthesis of terpenoids. The same was also observed when both moss and yeast *HMGR* was overexpressed in the α- and β-santalene producing lines (WT-STS6). Due to the low background production, the fold increase could not be determined for α/β-santalene, but the level of α- and β-santalene could be detected after the introduction. The use of moss or yeast *HMGR* did not show a difference in the levels of production, but would possibly allow for the use of both genes in future production lines in order to increase the yields even further. The increased levels of the sterols could allow for a down regulation of squalene synthase providing higher levels of FPP for sesquiterpenoid production. This was also done in yeast for sesquiterpenoid production using an inducible promoter (Ro et al., 2006).

The *CPS/KS* gene is the only terpene synthase gene in *P. patens* and catalyzes the first committed step in gibberellin biosynthesis converting GGPP to the precursor of gibberellins *ent*-kaur-16-ene (Hayashi et al., 2006; Zhan et al., in press). Thus, knockout of *CPS/KS* could lead to redirection of carbon flux toward the desired products since cross-talk between plastid and cytosol has been observed previously (Vranova et al., 2013). However, the yield of patchoulol, α- and β-santalene was not increased in any of the obtained knockout lines. The down-regulation of *PTS* itself could contribute to the reduced yield of patchoulol in PTS35-KO. However, the biosynthesis of stigmasterol was also decreased in KO lines, indicating occurrence of unknown regulatory events. Likewise, there are some indications that KO lines grow slower, though this needs further investigations.

Plastidic targeting of the sesquiterpenoid synthase is a unique metabolic engineering approach used in plants and has proved to be an effective strategy to increase patchoulol production in *N. tabacum* (Wu et al., 2006). Plastidic targeting of the PTS enzyme in *P. patens* did not afford higher yields, but significantly lower yields of patchoulol without the co-expression of an FPS enzyme. The limited FPP pool in the plastids of *P. patens* could be the reason for the lower yield. In order to compare the yield increase obtained in tobacco (40,000 fold) FPS would have to be targeted to the plastids as well. The little production of patchoulol in the plastidic PTS lines without a plastidic FPS do suggest that FPP is present endogenously in the plastids. FPP can be either formed by the incomplete condensation of IPP and DMAPP by GGPS, or transported from the moss cytosol (Hemmerlin et al., 2003; Laule et al., 2003; Hampel et al., 2005). In contrast to the plastid targeted PTS, *P. patens* with plastid targeted STS (WT-tpSTS1) showed a significant increase in α/β-santalene. The analysis of transcripts for *HMGR* and *STS* in this line showed that these genes were higher expressed (*STS* more than 1000-fold) than in the cytosolic STS lines, which cannot only be explained with the use of actin as reference gene. The higher expression resulted in the increased yields of α- and β-santalene from non-detectable level to 0.039 and 0.035 mg/g d.w. in *P. patens*.

An emission of monoterpenoids was observed in both the plastidic PTS and STS lines, showing that both enzymes are capable of utilizing geranyl pyrophosphate (GPP) as a substrate for enzymatic conversion. This is the first time that the PTS enzyme is reported to be capable of utilizing GPP to produce monoterpenoids in a plant host, whereas this functionality of the STS enzyme was previously reported in *in vitro* assays (Jones et al., 2011). The monoterpenoid profile observed here is clearly different from this publication indicating the different STS enzymatic properties toward GPP when studied *in vitro* or *in planta*. Further studies are needed to study the monoterpenoid functionality of the two sesquiterpene synthases.

Productivity of sesquiterpenoids in the transgenic PTS/STS lines is vital for the viability of *P. patens* as an industrial production platform. In our work, the moss lines were growing in shake flask condition and the biomass was accumulated from around 0.2 to 1 g/l d.w. in the liquid culture after 14 days, which was about 1/3 to 1/5 of the biomass titer obtained in an optimized bioreactor (cerff and posten, 2012). the highest productivity of patchoulol and α/β-santalene in our work was about 1.3 and 0.039 mg/g d.w. in 14 days, which is still too low for commercialization. however, there is a huge potential to use the rest of the endogenous isopentenyl pool to produce the desired products using metabolite engineering, as previously demonstrated in yeast. likewise, biomass accumulation through cultivation process optimization will also add to benefits of the moss *p. patens* as a production platform. in conclusion, the obtained results are promising for future development of *p. patens* as a biotechnological production host, and the capacity of the cells have not yet been reached.

### Conflict of interest statement

The authors declare that the research was conducted in the absence of any commercial or financial relationships that could be construed as a potential conflict of interest.

## References

[B1] Anterola A. Shanle E. Perroud P. F. Quatrano R. (2009). Production of taxa-4(5),11(12)-diene by transgenic *Physcomitrella patens*. Transgenic Res. 18, 655–660. 10.1007/s11248-009-9252-5 19241134

[B2] Asadollahi M. A. Maury J. Patil K. R. Schalk M. Clark A. Nielsen J. (2009). Enhancing sesquiterpene production in *Saccharomyces cerevisiae* through in *silico* driven metabolic engineering. Metab. Eng. 11, 328–334. 10.1016/j.ymben.2009.07.001 19619667

[B3] Asadollahi M. A. Maury J. Schalk M. Clark A. Nielsen J. (2010). Enhancement of farnesyl diphosphate pool as direct precursor of sesquiterpenes through metabolic engineering of the mevalonate pathway in *Saccharomyces cerevisiae*. Biotechnol. Bioeng. 106, 86–96. 10.1002/bit.22668 20091767

[B4] Bach S. S. King B. C. Zhan X. Simonsen H. T. Hamberger B. (2014). Heterologous stable expression of terpenoid biosynthetic genes using the moss *Physcomitrella patens*, in Plant Isoprenoids ed. Rodríguez-Concepción M. (New York, NY: Springer), 257–271. 10.1007/978-1-4939-0606-2_19 24777804

[B5] Berger R. G. (2007). Flavours and Fragrances Chemistry, Bioprocessing and Sustainability. Heidelberg: Springer.

[B6] Cerff M. Posten C. (2012). Relationship between light intensity and morphology of the moss *Physcomitrella patens* in a draft tube photo bioreactor. Biochem. Eng. J. 60, 119–126 10.1016/j.bej.2011.10.012

[B7] Chappell J. Wolf F. Proulx J. Cuellar R. Saunders C. (1995). Is the reaction catalyzed by 3-hydroxy-3-methylglutaryl coenzyme-a reductase a rate-limiting step for isoprenoid biosynthesis in plants. Plant Physiol. 109, 1337–1343. 12228673 10.1104/pp.109.4.1337 PMC157667

[B8] Cove D. J. Perroud P. F. Charron A. J. Mcdaniel S. F. Khandelwal A. Quatrano R. S. (2009). The moss *Physcomitrella patens*: a novel model system for plant development and genomic studies. Cold Spring Harb. Protoc. 2009:pdb.emo115. 10.1101/pdb.emo115 20147063

[B9] Cragg G. M. Kingston D. G. I. Newman D. J. (2011). Anticancer Agents from Natural Products. Boca Raton, FL: CRC Press.

[B10] Daviet J. Schalk M. (2010). Biotechnology in plant essential oil production: progress and perspective in metabolic engineering of the terpene pathway. Flavour Frag. J. 25, 123–127 10.1002/ffj.1981

[B11] Deguerry F. Pastore L. Wu S. Q. Clark A. Chappell J. Schalk M. (2006). The diverse sesquiterpene profile of patchouli, *Pogostemon cablin*, is correlated with a limited number of sesquiterpene synthases. Arch. Biochem. Biophys. 454, 123–136. 10.1016/j.abb.2006.08.006 16970904

[B12] Diaz-Chavez M. L. Moniodis J. Madilao L. L. Jancsik S. Keeling C. I. Barbour E. L.. (2013). Biosynthesis of sandalwood oil: *Santalum album* CYP76F cytochromes P450 produce santalols and bergamotol. PLoS ONE 8:e75053. 10.1371/journal.pone.0075053 24324844 PMC3854609

[B13] Drew D. P. Rasmussen S. K. Avato P. Simonsen H. T. (2012). A comparison of headspace solid-phase microextraction and classic hydrodistillation for the identification of volatile constituents from *Thapsia* spp. provides insights into guaianolide biosynthesi*s* in Apiaceae. Phytochem. Anal. 23, 44–51. 10.1002/pca.1323 21618308

[B14] Hampel D. Mosandl A. Wust M. (2005). Biosynthesis of mono- and sesquiterpenes in carrot roots and leaves (*Daucus carota* L.): metabolic cross talk of cytosolic mevalonate and plastidial methylerythritol phosphate pathways. Phytochemistry 66, 305–311. 10.1016/j.phytochem.2004.12.010 15680987

[B15] Hayashi K.-I. Horie K. Hiwatashi Y. Kawaide H. Yamaguchi S. Hanada A.. (2010). Endogenous diterpenes derived from ent-kaurene, a common gibberellin precursor, regulate protonema differentiation of the moss *Physcomitrella patens*. Plant Physiol. 153, 1085–1097. 10.1104/pp.110.157909 20488896 PMC2899919

[B16] Hayashi K. Kawaide H. Notomi M. Sakigi Y. Matsuo A. Nozaki H. (2006). Identification and functional analysis of bifunctional ent-kaurene synthase from the moss *Physcomitrella patens*. FEBS Lett. 580, 6175–6181. 10.1016/j.febslet.2006.10.018 17064690

[B17] Hemmerlin A. Hoeffler J. F. Meyer O. Tritsch D. Kagan I. A. Grosdemange-Billiard C.. (2003). Cross-talk between the cytosolic mevalonate and the plastidial methylerythritol phosphate pathways in tobacco bright yellow-2 cells. J. Biol. Chem. 278, 26666–26676. 10.1074/jbc.M302526200 12736259

[B18] Jones C. G. Moniodis J. Zulak K. G. Scaffidi A. Plummer J. A. Ghisalberti E. L.. (2011). Sandalwood fragrance biosynthesis involves sesquiterpene synthases of both the terpene synthase (TPS)-a and TPS-b subfamilies, including santalene synthases. J. Biol. Chem. 286, 17445–17454. 10.1074/jbc.M111.231787 21454632 PMC3093818

[B19] Laule O. Furholz A. Chang H. S. Zhu T. Wang X. Heifetz P. B.. (2003). Crosstalk between cytosolic and plastidial pathways of isoprenoid biosynthesis in *Arabidopsis thaliana*. Proc. Natl. Acad. Sci. U.S.A. 100, 6866–6871. 10.1073/pnas.1031755100 12748386 PMC164538

[B20] Livak K. J. Schmittgen T. D. (2001). Analysis of relative gene expression data using real-time quantitative PCR and the 2(-Delta Delta C(T)) method. Methods 25, 402–408. 10.1006/meth.2001.1262 11846609

[B21] Morikawa T. Saga H. Hashizume H. Ohta D. (2009). CYP710A genes encoding sterol C22-desaturase in *Physcomitrella patens* as molecular evidence for the evolutionary conservation of a sterol biosynthetic pathway in plants. Planta 229, 1311–1322. 10.1007/s00425-009-0916-4 19306103

[B22] Nørholm M. H. H. (2010). A mutant Pfu DNA polymerase designed for advanced uracil-excision DNA engineering. BMC Biotechnol. 10:21. 10.1186/1472-6750-10-21 20233396 PMC2847956

[B23] Nour-Eldin H. H. Geu-Flores F. Halkier B. A. (2010). USER cloning and USER fusion: the ideal cloning techniques for small and big laboratories. Methods Mol. Biol. 643, 185–200. 10.1007/978-1-60761-723-5_13 20552452

[B24] Nour-Eldin H. H. Hansen B. G. Norholm M. H. Jensen J. K. Halkier B. A. (2006). Advancing uracil-excision based cloning towards an ideal technique for cloning PCR fragments. Nucleic Acids Res. 34, e122. 10.1093/nar/gkl635 17000637 PMC1635280

[B25] Paddon C. J. Westfall P. J. Pitera D. J. Benjamin K. Fisher K. Mcphee D.. (2013). High-level semi-synthetic production of the potent antimalarial artemisinin. Nature 496, 528–532. 10.1038/nature12051 23575629

[B26] Pan X.-W. Han L. Zhang Y.-H. Chen D.-F. Simonsen H. T. (in press). Biotechnological production of sclareol in the moss Physcomitrella patens. Front. Plant Sci.

[B27] Ro D. K. Ouellet M. Paradise E. M. Burd H. Eng D. Paddon C. J.. (2008). Induction of multiple pleiotropic drug resistance genes in yeast engineered to produce an increased level of anti-malarial drug precursor, artemisinic acid. BMC Biotechnol. 8:83. 10.1186/1472-6750-8-83 18983675 PMC2588579

[B28] Ro D. K. Paradise E. M. Ouellet M. Fisher K. J. Newman K. L. Ndungu J. M.. (2006). Production of the antimalarial drug precursor artemisinic acid in engineered yeast. Nature 440, 940–943. 10.1038/nature04640 16612385

[B29] Scalcinati G. Partow S. Siewers V. Schalk M. Daviet L. Nielsen J. (2012). Combined metabolic engineering of precursor and co-factor supply to increase alpha-santalene production by *Saccharomyces cerevisiae*. Microb. Cell Fact. 11, 117. 10.1186/1475-2859-11-117 22938570 PMC3527295

[B30] Simonsen H. T. Drew D. P. Lunde C. (2009a). Perspectives on using *Physcomitrella patens* as an alternative production platform for thapsigargin and other terpenoid drug candidates. Perspect. Medicin. Chem. 3, 1–6. 19812738 10.4137/pmc.s2220 PMC2754923

[B31] Simonsen H. T. Riedel C. Gade L. B. Jebjerg C. P. Guzman A. Mølgaard P. (2009b). Chemical composition and antibacterial activity of the leaf essential oil of *Baccharis magellanica* (Lam.) Pers. and *Baccharis elaeoides* Remy from Chile. J. Essent. Oil Res. 21, 377–380 10.1080/10412905.2009.9700196

[B32] Simonsen H. T. Weitzel C. Christensen S. B. (2013). Guaianolide sesquiterpenoids - their pharmacology and biosynthesis, in Handbook of Natural Products - Phytochemistry, Botany and Metabolism of Alkaloids, Phenolics and Terpenes, eds Ramawat K. G. Merillon J. M. (Berlin: Springer-Verlag), 3069–3098.

[B33] Stenøien H. K. (2004). Adaptive basis of codon usage in the haploid moss *Physcomitrella patens*. Heredity 94, 87–93. 10.1038/sj.hdy.6800547 15483656

[B34] Stenøien H. K. (2007). Compact genes are highly expressed in the moss *Physcomitrella patens*. J. Evol. Biol. 20, 1223–1229. 10.1111/j.1420-9101.2007.01301.x 17465932

[B35] Von Schwartzenberg K. Schultze W. Kassner H. (2004). The moss *Physcomitrella patens* releases a tetracyclic diterpene. Plant Cell Rep. 22, 780–786. 10.1007/s00299-004-0754-6 14963693

[B36] Vranova E. Coman D. Gruissem W. (2013). Network analysis of the MVA and MEP pathways for isoprenoid synthesis. Annu. Rev. Plant Biol. 64, 665–700. 10.1146/annurev-arplant-050312-120116 23451776

[B37] Weitzel C. Simonsen H. (2013). Cytochrome P450-enzymes involved in the biosynthesis of mono- and sesquiterpenes. Phytochem. Rev. 1–18 10.1007/s11101-013-9280-x

[B38] Wu S. Schalk M. Clark A. Miles R. B. Coates R. Chappell J. (2006). Redirection of cytosolic or plastidic isoprenoid precursors elevates terpene production in plants. Nat. Biotechnol. 24, 1441–1447. 10.1038/nbt1251 17057703

[B39] Zhan X. Bach S. S. Lunde C. King B. C. Simonsen H. T. (in press). Three additional diterpenes from Pyscomitrella patens synthesized by copalyl diphosphate/kaurene synthase (PpCPS/KS). Phytochemistry. 10.1016/j.plaphy.2015.07.011 26248039

[B40] Zwenger S. Basu C. (2008). Plant terpenoids: applications and future potentials. Biotechn. Mol. Biol. Rev. 3, 1–7.

