# Supplementary material for: Metabolic engineering of the moss Physcomitrella patens to produce the sesquiterpenoids patchoulol and α/β-santalene
Source: Front Plant Sci. 2014 Nov 18;5:636. doi: 10.3389/fpls.2014.00636 (PMC4235272; doi:10.3389/fpls.2014.00636)
Supplement: Supplementary file 1 [file Table1.DOCX]

**Supporting information Table S1-S3**

Table S1. Nucleotide sequences of the primers used for vectors construction

| Primer | Nucleotide seuqnece (5’->3’) |
| --- | --- |
| PTS-F | GGCTTAAUATGGAGTTGTATGCCCAAAG |
| PTS-R | GGTTTAAUTTAATATGGAACAGGGTGAAG |
| STS-F | GGCTTAAUATGGATTCTTCCACCGCCAC |
| STS-R | GGTTTAAUCTACTCCTCGCCGAGAGGAA |
| PptHMGR-F | GGCTTAAUATGTTCATCGGCAAGAGCGGTGAT |
| PptHMGR-R | GGTTTAAUTCAGGCGGAAGTAGTCTTAGTTG |
| SctHMGR-F | GGCTTAAUATGGCTGCAGACCAATTGGTG |
| SctHMGR-R | GGTTTAAUTTAGGATTTAATGCAGGTGACGGA |
| tp-F | GGCTTAAUATGGCTTCCTCTATGCTCTCCTC |
| PTS-R | GGTTTAAUTTAATATGGAACAGGGTGAAGGTACAAC |
| 2A-tpFPS-F | AGTTGGCAGGAGAUGTGGAATCTAACCCAGGACCTATGGCTTCCTCTATGCTCTCCTC |
| FPS-R | GGTTTAAUTCATTTCTGGCGTTTGTAGATCTTC |
| tp-R | AGATCTUCCCCCGTTGCTTGC |
| tp-STS-F | AAGATCUAGCTGCATGAAGGAGCTCGGCGCGCCTATGGATTCTTCCACCGCCACC |
| oCL252 | CTCTGTCTCTCCCACAATCCTCC |
| oCL77 | CCTGTGCAAGGTAAGAAGATGG |
| oSSB94 | AAGCCGACTTCAACATGTG |
| oSSB95 | AACCCGAAGCTCTTCCAC |

Table S2. Vectors used and constructed for PEG-mediated moss transformation

| vector | description |
| --- | --- |
| pCAMBIA230035Su | pCAMBIA2300 with USER cassette, hygromycin resistant |
| pCAMBIA130035Su | pCAMBIA1300 with USER cassette, kanamycin resistant |
| pUNI33 | pJET1.2 backbone with the essential compartment from pCAMBIA130035Su (plant antibiotic resistant cassette, 35S promoter-USER cloning site-35S terminator) |
| pUNI6 | pJET1.2 backbone with the essential compartment from pCAMBIA230035Su (plant antibiotic resistant cassette, 35S promoter-USER cloning site-35S terminator) |
| pUNI33 PTS | pUNI33 containing the coding region of the PTS gene |
| pUNI33 STS | pUNI33 containing the coding region of the STS gene |
| pUNI33 tpPTS | pUNI33 containing the PTS gene with transit peptide sequence of Arabidopsis RuBisCO small subunit |
| pUNI33 tpSTS | pUNI33 containing the STS gene with transit peptide sequence of Arabidopsis RuBisCO small subunit |
| pUNI33 tpPTS-2A-tpFPS | pUNI33 containing the plastidic-targeting PTS and plastidic-targeting FPS gene with 2A linker |
| pUNI6 PptHMGR | pUNI6 containing the truncated *P. patens* HMGR gene with only the catalytic domain |
| pUNI6 SctHMGR | pUNI6 containing the truncated *S. cerevisiae* HMGR gene with only the catalytic domain |
| pCL755 | pDONR201::CPS/KS containing a cassette containing p35S-nptII-CamVter between two 1,5 kb homologous recombination arms from the CPS/KS gene |

Table S3. The selected nucleotide sequences of the qPCR primers

| gene | Primer | Nucleotide sequence |
| --- | --- | --- |
| PTS | PTS-QF | AAGCACAAACCCACAACCAAGGAG |
|  | PTS-QR | AGAGCTTACATGGAAGAGGCCCAA |
| STS | STS-QF | CCTTCCTGATCTTCTGCACTAC |
|  | STS-QR | ATTATCGCCTCTTGCCATCTC |
| PpHMGR | P100 | CCATTCGTCCAACATTTTGATGA |
|  | P101 | GGGAGCAAGCAAGTTTAACACTG |
| PptHMGR | PptHMGR-QF | GGCTGGTGAGATCTCTCTTATG |
|  | PptHMGR-QR | CTTAGTTGGTAGAGGAGGCAAG |
| SctHMGR | SctHMGR-QF | CCGTCTTGGCAGGTGAATTA |
|  | SctHMGR-QR | GGTTTCCTGTTGTGGGTCATA |
| Actin2 | P84 | GCGAAGAGCGAGTATGACGAG |
|  | P85 | CTCCATAACCCCACCTGACAA |
