# Supplementary material for: Metabolic engineering of the moss Physcomitrella patens to produce the sesquiterpenoids patchoulol and α/β-santalene
Source: Front Plant Sci. 2014 Nov 18;5:636. doi: 10.3389/fpls.2014.00636 (PMC4235272; doi:10.3389/fpls.2014.00636)

1. β-patchoulene RI=1389

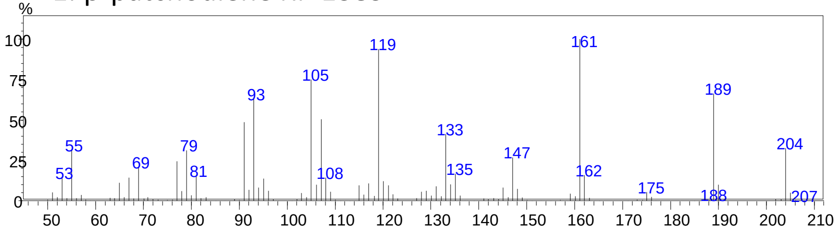

2. β-caryophyllene RI=1428

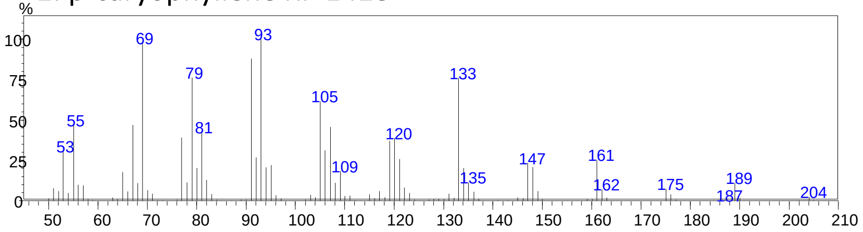

3. α-guaiene RI=1447

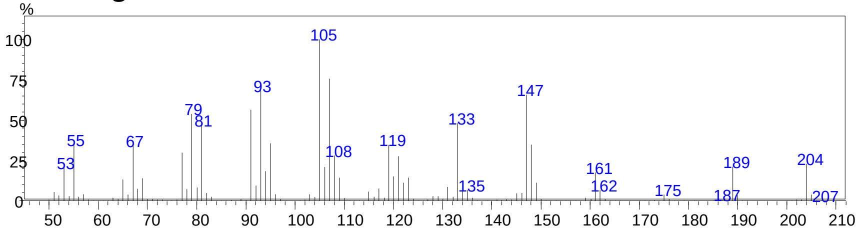

4. seychellene RI=1453

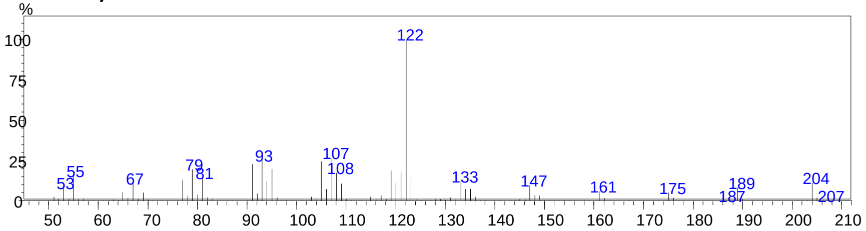

5. α-patchoulene RI=1467

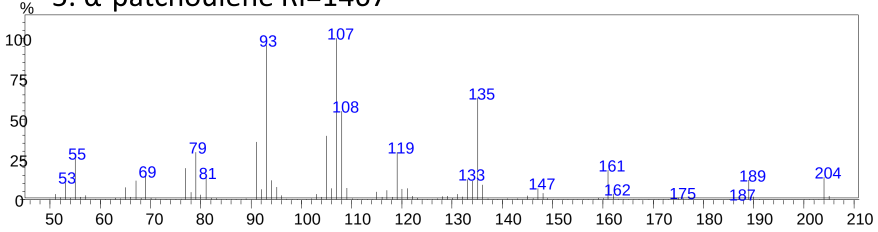

6. γ-patchoulene RI=1472

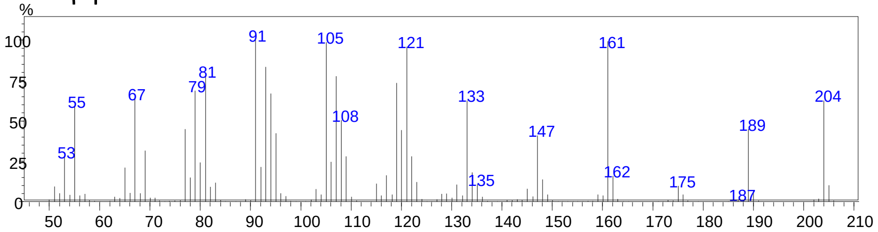

7. guai-4,11-diene RI=1485

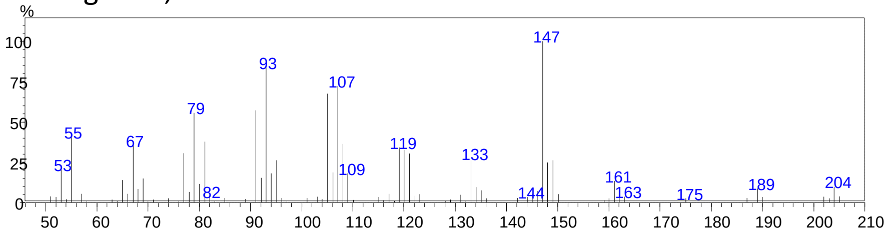

8. α-selinene RI=1497

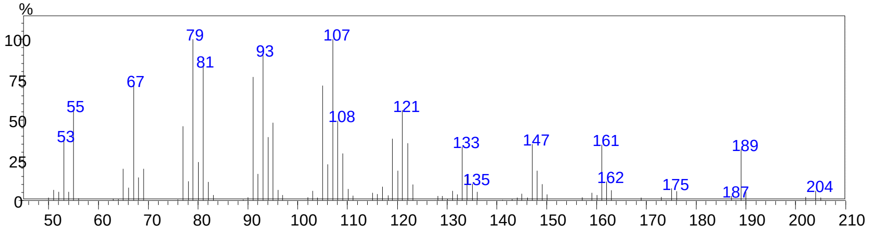

9. δ-guaiene RI=1514

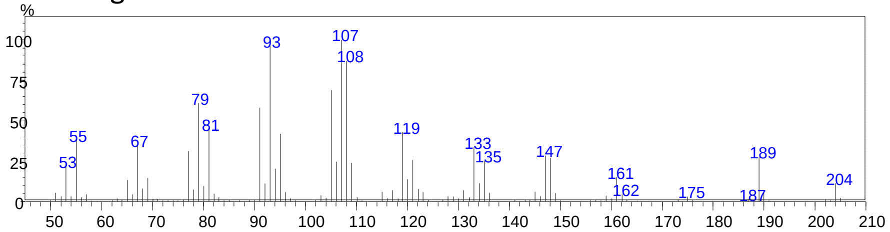

10. α-panasinsen RI=1527

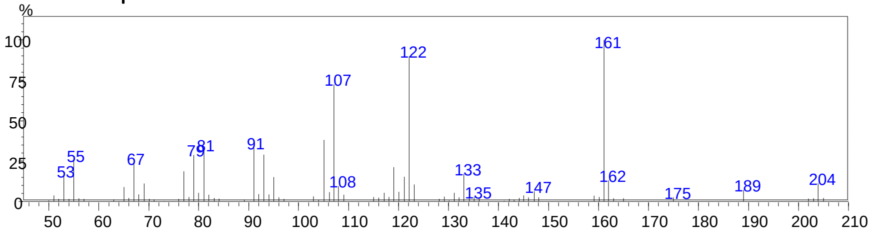

11. patchoulol RI=1672

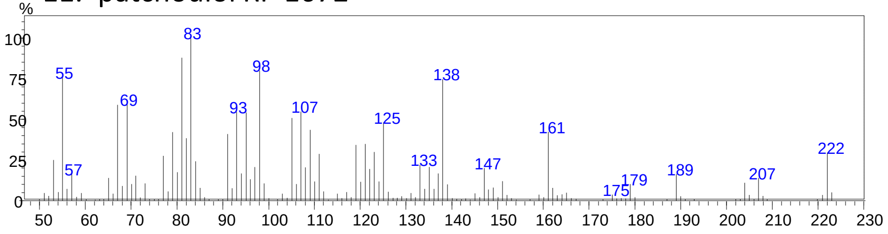

12. β-myrcene RI=990

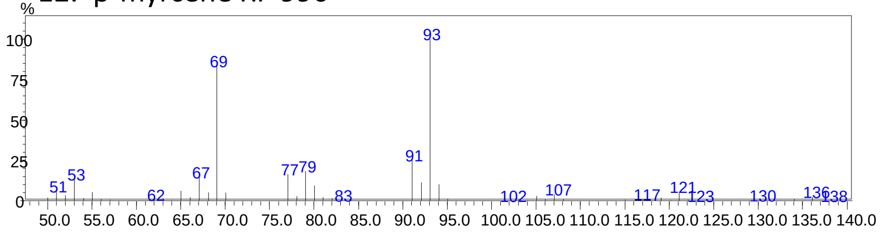

13. limonene RI=1028

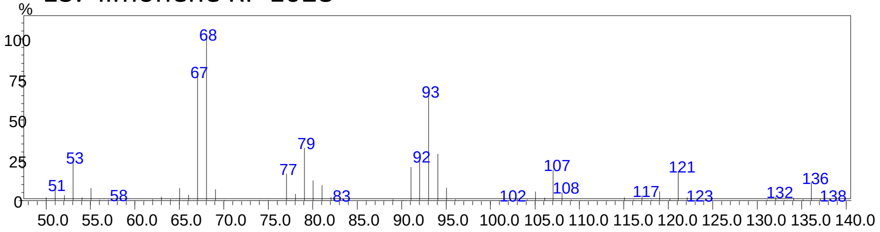

14. γ-terpinene RI=1060

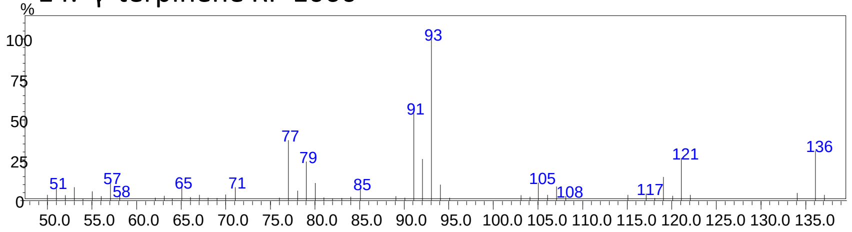

15. α-terpinolene RI=1089

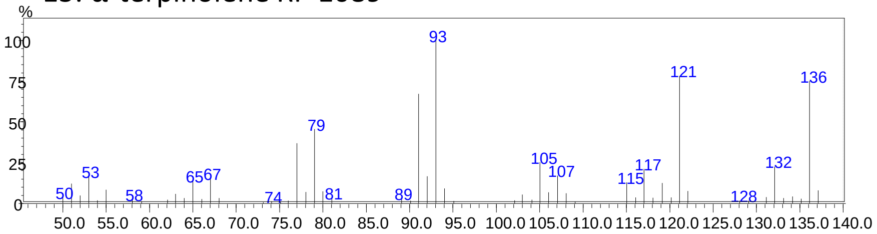

21. α-santalene RI=1426

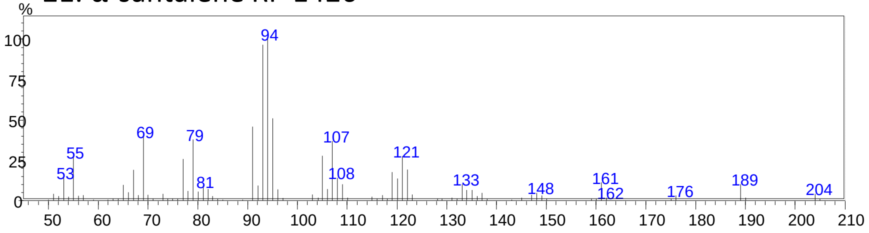

22. (E)-α-bergamotene RI=1442

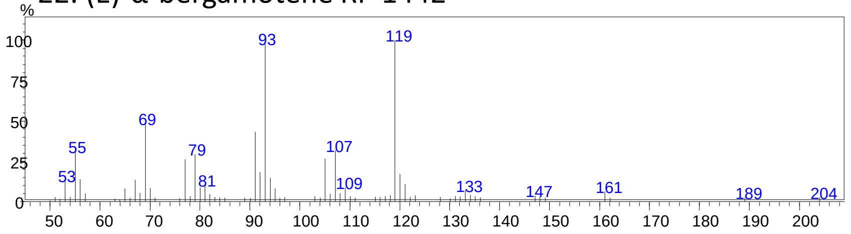

23. epi-β-santalene RI=1454

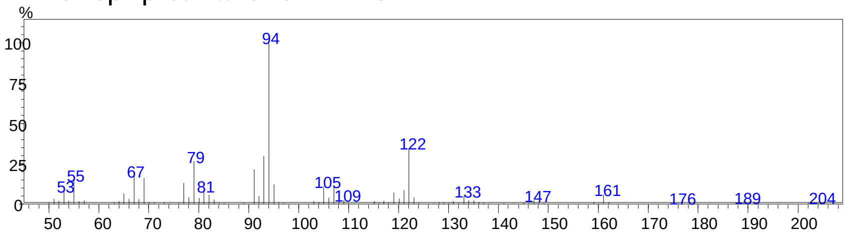

24. β-santalene RI=1467

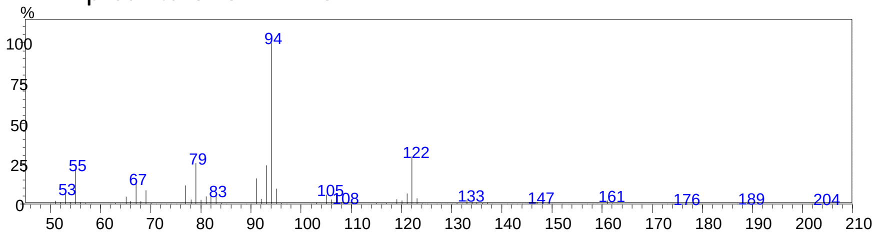

25. α-pinene RI=927

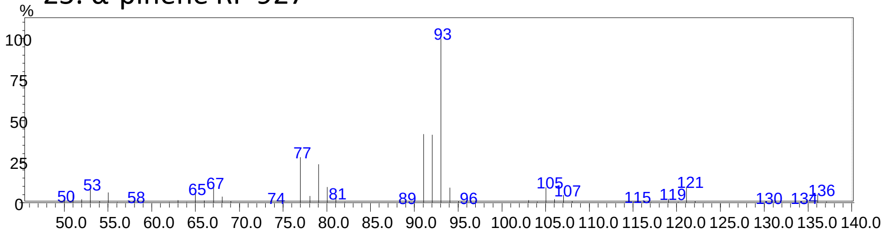

26. camphene RI=942

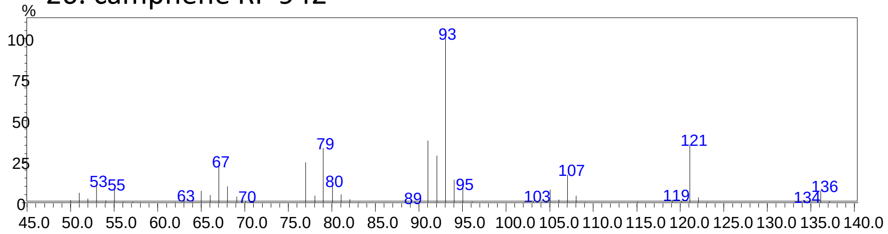

27. β-pinene RI=973

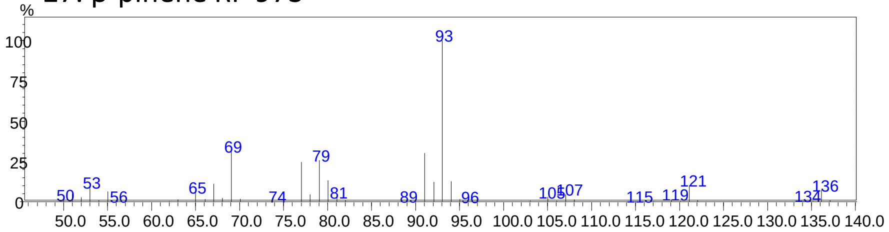

28. linalool RI=1100

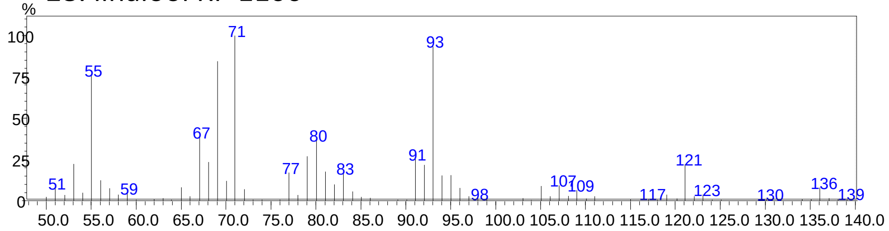

29. (E)-β-farnesene RI=1492

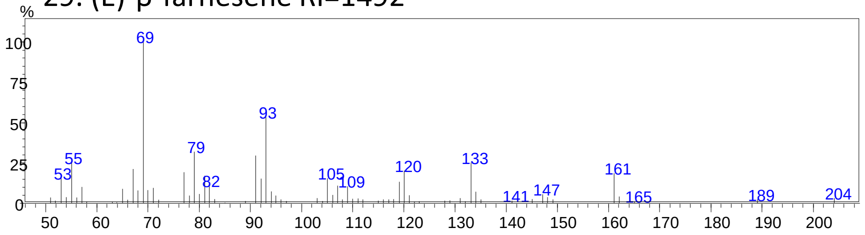

30. β-bisabolene RI=1514

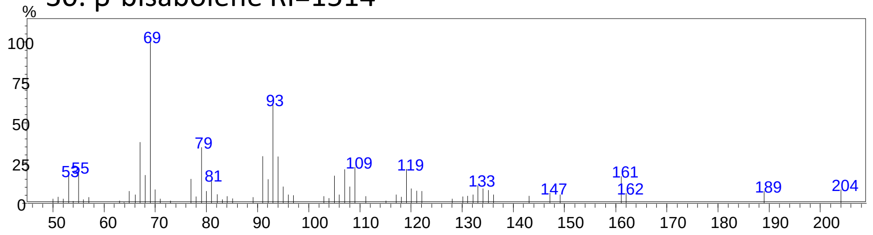

31. α-santalan-10-ol RI=1594

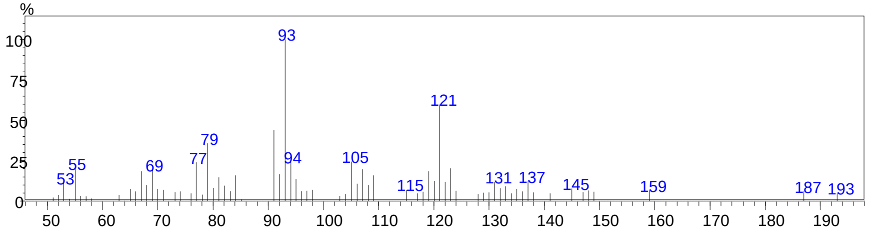

authentic patchoulol RI=1672

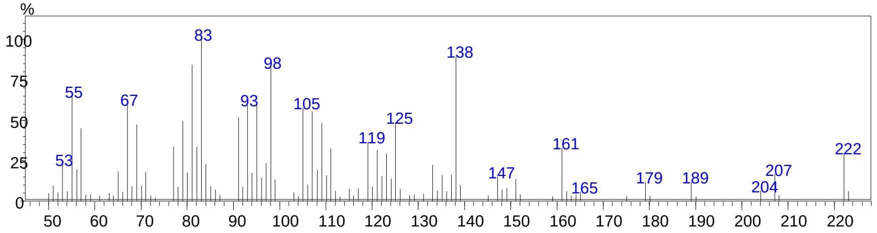

authentic α-santalene RI=1426

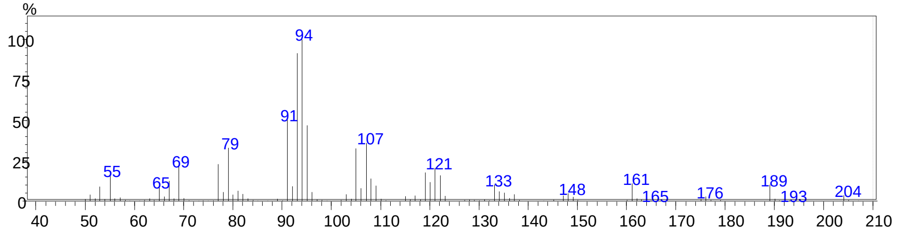

Supplement: Supplementary file 2 [file Image1.PDF]
